# Transplacental Transfer of Primaquine and Neurobehavioral Development of Prenatally Exposed Rats

**DOI:** 10.1155/2021/7392606

**Published:** 2021-06-24

**Authors:** Klaus M. Becker, Lorenna Rosa, Manoella S. B. Fernandes, Rosangela R. de Carvalho, Ana Cecilia X. De-Oliveira, Davyson L. Moreira, Francisco J. R. Paumgartten

**Affiliations:** ^1^Department of Biological Sciences, National School of Public Health (FIOCRUZ), Rio de Janeiro 21040-361, Brazil; ^2^Institute of Drug Technology (Farmanguinhos), Oswaldo Cruz Foundation (FIOCRUZ), Rio de Janeiro 22.775-610, Brazil

## Abstract

Primaquine (PQ) not only eliminates *P. falciparum* gametocytes but also kills liver dormant forms of *P. vivax* and *P. ovale*. Owing to these unique therapeutic properties, it is an essential drug. Although PQ has been used for over 70 years, its toxicological database has gaps such as the absence of studies on its reproductive and developmental toxicity and kinetics in pregnancy. This study investigated the transplacental transfer of PQ and the effects of intrauterine exposure on the postnatal growth, survival, and neurobehavioral development of the offspring. PQ kinetics and transplacental transfer were investigated in rats treated orally (40 mg.kg·bw^−1^) on gestation day (GD) 21. PQ was analyzed by high-performance liquid chromatography with diode array ultraviolet detection. To evaluate effects of intrauterine exposure on postnatal development, dams were treated orally with PQ (20 mg.kg·bw^−1^·d^−1^) or water (controls) on GD 0–21. Postnatal survival, body weight gain, somatic maturation, and reflex acquisition were evaluated. The open field test (OF) was conducted on PND 25. PQ concentration in the fetal plasma was nearly half that in maternal plasma. Except for increase in pregnancy loss, no effects of PQ were noted at term pregnancy and first days of life. Prenatal PQ did not affect postnatal weight gain nor did it impair somatic and neurologic development of the offspring. Pups born to PQ-treated dams showed reduced exploration and enhanced emotionality in the OF. PQ given in pregnancy, at doses greater than those recommended for malaria therapy, may affect pup postnatal survival and emotional behavior.

## 1. Introduction

Primaquine (PQ), an 8-aminoquinoline derivative introduced in the early 1950s, plays a key role in the current therapeutic armamentarium for management of malaria. It is not only a gametocytocidal drug for *P. falciparum* infections, but also effective in killing hypnozoites, liver dormant forms of *P. vivax* and *P. ovale* parasites. The 8-aminoquinoline drugs (*e.g*., PQ and tafenoquine) when combined to schizonticidal antimalarials eradicate *P. vivax* parasites in patients (a radical cure) thereby preventing their recurrence [1–4]. Owing to its unique therapeutic properties (transmission-blocking in falciparum malaria, and prevention of relapses in *P*. *vivax* infections), the World Health Organization (WHO) listed PQ among the essential medicines [5].

In clinical use for 70 years or so, PQ proved to be well tolerated by most patients. Its adverse effects are generally mild and restricted to symptoms of gastrointestinal discomfort such as nausea, vomiting, dizziness, stomach upset, abdominal cramps, and weakness. A rise in metahemoglobinemia levels of no/minor clinical relevance may occur as well [6]. In glucose-6-phosphate dehydrogenase (G6PD) deficient patients, however, PQ triggers a severe and sometimes fatal acute hemolytic anemia. In fact, PQ exerted a pivotal role in the discovery of a link between inborn G6PD deficiencies and hemolytic responses to certain drugs [7].

G6PD deficiency, the second most common enzyme deficiency in humans, is a chromosome X-linked recessive disorder [8]. The enzyme disorder is asymptomatic until G6PD-deficient people are exposed to factors that trigger acute hemolytic anemia symptoms. Because G6DP participates in the defense of red blood cells (RBCs) against oxidative damage, drugs (e.g., primaquine and dapsone) and other conditions that enhance intracellular oxidative stress (e.g., certain infectious diseases, and fava beans) may elicit hemolytic reactions in susceptible individuals. In newborns, acute hemolytic anemia may result in neonatal pathological jaundice and kernicterus, a rare life-threatening complication.

Like any other drug with potential to cause fetal or neonatal hemolytic anemia, PQ must not be used in pregnancy. In principle, a diagnostic test for G6PD status should allow avoiding PQ-induced maternal hemolytic anemia [9]. Nonetheless, even if the mother is G6PD-normal (heterozygous), her unborn male or female child might not be. Although being possible with modern DNA-technology and amniocentesis, the prenatal diagnosis of G6PD status is not practical nor is aminocentesis devoid of risks for the fetus [10]. To protect the unborn child, PQ has been strictly contraindicated in pregnancy since mid-1950s. By virtue of this longstanding contraindication, there is no clinical data on the pharmacokinetics and/or safety of PQ during pregnancy [11].

Whether PQ could be safely used in pregnancy was not adequately investigated by nonclinical studies either. PQ began to be used as antimalarial drug before the guidelines for nonclinical safety studies (including a comprehensive evaluation of reproductive toxicity) were introduced in the aftermath of the thalidomide tragedy. Since then, the fact that PQ was contraindicated for use in pregnancy has discouraged any further in-depth investigation of its developmental toxicity [11].

Data on the effects of PQ in pregnant animals are scanty [11]. Pregnancy losses and fetal abnormalities at doses causing severe maternal toxicity and deaths were reported by two studies in which rats were treated orally with PQ (10.3, 30.8, and 61.5 mg.kg·bw^−1^·d^−1^ and 0.57, 5.7, 11.4, and 34 mg.kg·bw^−1^d^−1^) on pregnancy days 6–15 and 8–16, respectively [12, 13]. Another study described that PQ (0.25 to 3.0 mg.kg·bw^−1^·d^−1^) administered to rats on GD 0–20 caused no harmful effects on the mothers and their offspring [14]. The data from these three studies, however, are limited and far from being enough for a thorough assessment of reproductive and developmental risks of PQ.

It is of note that severe neurological side effects were noted with some of the first 8-aminoquinolines used in clinical practice. Although PQ and tafenoquine have been reported not to cause neurologic adverse effects at therapeutic doses [4, 15], a possible neurodevelopmental toxicity of these antimalarials has not been assessed so far.

This study was undertaken to investigate the kinetics and transplacental transfer of PQ in pregnant rats, and, also, the postnatal growth, survival, and neurobehavioral development of prenatally exposed offspring.

## 2. Materials and Methods

### 2.1. Chemicals

The chemicals were of analytical reagent grade or a higher purity (HPLC-grade). The solvents (methanol and acetonitrile) were supplied by Tedia® (Rio de Janeiro, Brazil). Ammonium acetate (CAS 631- 61-8, CH_3_COONH_4_), zinc sulfate heptahydrate (ZnSO_4_·7H_2_O), and acetic acid (CAS 64-19-7, CH_3_COOH) were from Merck Millipore®. Sodium heparin (5,000 IU·mL^−1^) was purchased from Hipolabor (Belo Horizonte, Brazil). Ultrapure water was provided daily by a Milli-Q® purifying water system (Merck-Millipore).

### 2.2. Animals

Male and nulliparous female Wistar rats (aged 70–90 days) were supplied by the Oswaldo Cruz Foundation Central Animal House (FIOCRUZ, Rio de Janeiro, Brazil). All rats were housed in standard plastic cages with stainless steel covers and white pine shavings as bedding and were maintained under controlled environmental conditions (12-h light:12-h dark photoperiod, lights on from 7:00 a.m. to 7:00 p.m.; room temperature 20 ± 1°C; relative air humidity approximately 70%). An autoclaved pellet diet for rats and mice (Nuvital, Nuvilab Ltd., Curitiba, PR, Brazil) and filtered tap water were available ad libitum. The experimental protocol was approved by the Ethical Committee on the Use of Laboratory Animals of FIOCRUZ (CEUA-FIOCRUZ; project licence numbers LW11/19 and LW22/19). A preliminary single-dose toxicity test of PQ was conducted for choosing the doses to be used in pharmacokinetic and developmental toxicity experiments (see Results section).

### 2.3. Mating Procedure

Mating was accomplished by transferring two females into a male's cage for 2 h at the end of the dark period. The day on which copulation was confirmed by the presence of spermatozoa in the vaginal smear was designated as gestation day 0 (GD 0).

### 2.4. Treatment

The rats were treated with freshly prepared PQ solutions in ultrapure water (2 mg of PQ base·mL^−1^ equivalent to 3.551 mg of PQ diphosphate·mL^−1^). Doses and concentrations are expressed in terms of PQ base. PQ diphosphate (Genix Indústria Farmacêutica Ltda (PP3016PQRJ, 98–102%; Anápolis, Brazil) was administered by oral gavage at doses equivalent to 20 or 40 mg.kg·bw^−1^. Controls received equal volumes of the vehicle (ultrapure water) only. The volume administered by gavage was 6 mL·kg·bw^−1^. To evaluate the effects of in utero exposure on the development of the offspring after birth, dams were daily treated with PQ (20 mg.kg·bw^−1^·d^−1^, p.o.) on GDs 0–21. The kinetics of PQ (20 and 40 mg.kg·bw^−1^) in pregnancy and lactation was evaluated in a separate group of females treated with a single oral dose on GD21 (transplacental transfer), or on PND 19 (transfer to pups via maternal milk).

### 2.5. Analysis of Primaquine (PQ) in Blood Plasma

Concentrations of PQ in the blood plasma were analyzed by a high-performance liquid chromatography (HPLC) method developed at our laboratory [16]. In brief, after plasma protein precipitation, PQ was analyzed by HPLC-DAD-UV with a modified-silica cyanopropyl column (250 mm × 4.6 mm i.d. × 5 *μ*m) as the stationary phase and a mixture of acetonitrile and 10 mM ammonium acetate buffer (pH = 3.80) (45 : 55) as the mobile phase. The flow rate was 1.0 mL·min^−1^, the oven temperature was 50°C, and absorbance was measured at 264 nm. Limits of detection (LOD) and quantification (LOQ) were 1.0 and 3.5 ng·mL^−1^, respectively.

### 2.6. Evaluation of Somatic and Neurobehavioral Development

From GD20 onwards, dams' cages were checked twice a day for spontaneous deliveries. On the day of birth (PND 1), viable and dead neonates were counted in each litter, and pups were sexed, weighed, and individually marked (India ink tattoo). Pup body weight was recorded on PNDs 1, 5, 10, 15, 20, and 30. Litters were weaned on PND 24.

All pups were examined for determining the postnatal day on which appeared landmarks of somatic maturation and neurological reflexes were acquired. During the assessment, and prior to testing each pup, the mother was placed in a holding cage. Until postnatal day 14, pups were kept in a warm environment (37°C) from where they were removed individually for testing.

The following somatic maturation landmarks were assessed:

  Incisor eruption: eruption of the upper and lower incisors through the gums.
  Fur development: downy hair was first detected.
  Ear unfolding: any detachment of the external pinnae of both ears.
  Eye opening: total separation of the upper and lower eyelids and complete opening of both eyes.
  Vaginal opening: discontinuity of the skin and appearance of a definite aperture in the vaginal area
  Descent of testes: testicular descent (both testes) confirmed by scrotum palpation.
  Preputial separation: separation of the prepuce from the glans penis assessed by attempting to retract the prepuce. The day of complete preputial separation was the endpoint used in the analysis.

The appearance (or loss) of neurological reflexes development was tested as follows:

  Surface righting: the pup was placed on back on a flat surface, and the time for it to turn over to rest in the prone position with all four feet on the ground was recorded. The response was positive when pup had turned over to the prone position within 30 s.
  Cliff avoidance: the pup was placed on the edge of a bench with its nose and forefeet just over the edge. A response was considered positive when the pup moved away from the cliff in less than 60 s.
  Negative geotaxis: the pup was placed head downwards on a 40° slope, and the time for it to turn 180° to face up the slope was recorded. A response was positive when this movement occurred in less than 60 s.
  Auditory startle response: a spring-loaded metal rat trap held above and behind the pup was closed. A positive response was indicated by a whole-body startle in response to the sound stimulus.
  Palmar grasp: a pup forepaw was gently stroked with a paper clip, and the digit flexing response was observed. The day on which the flexing response disappeared was recorded.
  Free-fall righting: the pup was dropped, back downwards, from a height of 30 cm onto a cotton wool pad. Turning in midair and landing on the surface in a fully prone position was a positive response. Two perfect landings out of three tests on one day was the criterion for reflex acquisition.

The postnatal days at which pups began to be examined or tested were as follows: PND 1: ear unfolding, fur development surface righting, palmar grasp, and negative geotaxis; PND 2: cliff avoidance; PND 6: incisor eruption; PND 12: eye opening, free-fall righting, and auditory startle response; and PND 14: testes descent; PND 30: preputial separation and vaginal opening.

### 2.7. Open Field Test

The open field (OF) apparatus (a circular arena) was described in detail elsewhere [17]. Each rat was placed on the central circle of the OF and its behavior was observed for a period of 6 minutes. The exploratory activity was scored at three (2 min) time intervals as follows: latency (time to leave the central circle), locomotor activity (number of floor subdivisions traversed), rearing up (number of times the animal stood on its two hind legs), grooming (number of grooming behavior episodes), and fecal boli (number of fecal boli left on the arena floor). The arena was cleaned with ethanol solution (70%) and allowed to dry after testing each rat. The open field test was conducted when pups were 25 days old.

### 2.8. Statistical Analysis

Whenever data refer to the offspring of pregnant rats, the litter and not the individual fetus/pup was the statistical unit of analysis. Comparisons between two group means were made by Student's *t*-test or by the Mann–Whitney *U* test, whenever data are likely not to exhibit a normal distribution. Proportions were compared by the chi-square test or Fisher's exact test. The statistical calculations were made using a statistical software (Graph Pad Prism 5.0.). Differences were considered significant when *p* < 0.05. Data on the day of appearance of developmental landmarks are shown as the group median value and range (minimum–maximum), whereas all other data are represented as means ± SD.

## 3. Results

### 3.1. Preliminary Dose Selection Test: PQ Single-Dose Toxicity

Owing to the paucity of nonclinical toxicity data on PQ, a preliminary test in female rats was undertaken to choose doses and routes of administration to be used in this study. As indicated in Table 1, rats receiving single oral doses of PQ as high as 40 (*N* = 2) and 30 mg.kg·bw^−1^ (*N* = 2) survived to treatment exhibiting only transient symptoms such as hypoactivity and teeth grinding during the 24 h observation period. All rats receiving doses of PQ higher than 5 mg.kg·bw^−1^ by intravenous injections, however, died within a few minutes of the treatment showing neuromuscular symptoms such as hyperactivity, muscle spasms, and tremors preceding coma and death. Based on these toxicity data, an oral dose of 20 mg.kg·bw^−1^·d^−1^ was selected for repeated dosing during whole pregnancy and a single dose of 40 mg.kg·bw^−1^ (oral) for acute pharmacokinetic determinations on GD 21.

### 3.2. Pharmacokinetics in Pregnancy and Transplacental Transfer

**Figure 1:**
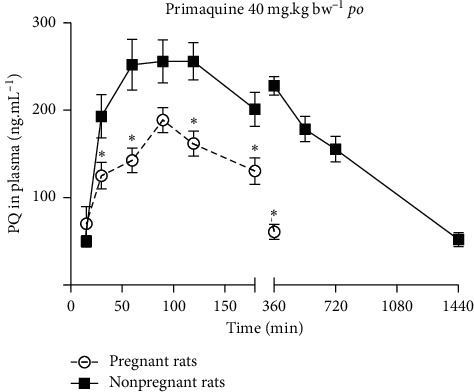
Primaquine (PQ) disposition in nonpregnant and pregnant rats: plasma concentration-time curves for PQ (40 mg.kg·bw^−1^) administered by the oral (gavage) route (*N* = 10 per group). ^*∗*^*p* < 0.05.

Following the oral administration to nonpregnant rats, PQ was rapidly absorbed from the GI tract reaching plasma maximum levels (*C*_max_) within 60 to 120 minutes of treatment (Figure 1).

**Figure 2:**
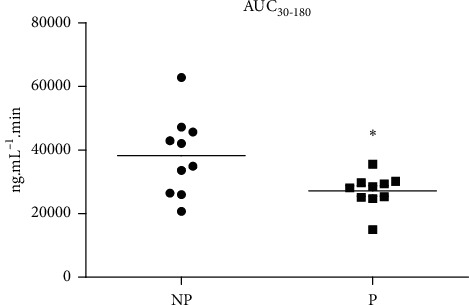
Primaquine (PQ) disposition in nonpregnant (NP) and pregnant (P) rats: areas under the curve (AUC_30-180_) for PQ (40 mg.kg·bw^−1^) administered by the oral route (gavage) on GD 21. ^*∗*^(Student's *t*-test): *p* < 0.05.

In pregnant rats, oral administration of PQ gave rise to plasma levels of this antimalarial drug lower than those achieved in nonpregnant females (Figures 1 and 2). Moreover, the areas under the curve (AUCs) for plasma concentration of PQ versus time in near term pregnant rats were smaller than respective AUCs in nonpregnant animals (Figure 2).

Pharmacokinetic changes in pregnancy, particularly during the third trimester or near term, have been described by human clinical studies and may result from anatomical and physiological alterations taking place in the gravid female organism [18]. Pregnancy associated increases in volume distribution (Vd) and/or presystemic clearance (first-pass metabolism) of PQ are possible explanations for AUC changes in term pregnancy.

**Figure 3:**
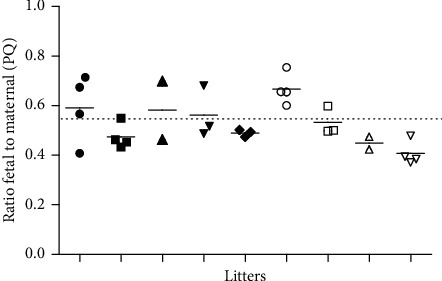
Transplacental transfer of primaquine (PQ). Ratios of fetal to maternal plasma concentrations of PQ in pregnant rats treated orally with PQ (40 mg.kg·bw^−1^). Samples of maternal and fetal blood (2 to 4 fetal blood pooled samples per litter) were drawn 180 min after PQ administration.

Data from this study corroborate the notion that PQ in the maternal blood is largely transferred across the placenta to the embryofetal compartment. The mean concentrations of PQ in the maternal (*N* = 9) and fetal (*N* = 9 means of litter, means ± SD) plasma were 170.2 ± 47.8 and 89.4 ± 25.1 ng·mL^−1^, respectively. As shown in Figure 3, the ratios of fetal to maternal plasma concentrations are approximately 0.5 (mean ± SD = 0.54 ± 0.09) and did not vary significantly among different mother/litters (Kruskal–Wallis test *p* > 0.05).

### 3.3. Transfer of PQ from Nursing Females to Their Pups

Nursing mothers are generally recommended not to breastfeed their infants while taking PQ for malaria treatment. A recent clinical trial, however, showed that PQ was poorly excreted into breastmilk and undetectable in the blood of breastfeeding infants [19]. In this study we noted that, in all pups from the two tested litters (PND19), plasma levels of PQ were below the detection limit of the method (<1.0 ng·mL^−1^) 2 and 4 hours after it was administered (20 mg.kg·bw^−1^, p.o.) to their nursing mothers. Data from this experimental study, therefore, are consistent with the reported clinical data.

### 3.4. Postnatal Survival, Somatic Maturation, and Neurobehavioral Development

No behavioral changes or other signs of toxicity were noted among PQ-treated dams during the whole pregnancy. Pregnancy weight gain (GD21-GD0) was unaffected by maternal treatment with PQ (20 mg.kg·bw^−1^·d^−1^) on GDs 0–21 (Table 2). The length of pregnancy (number of days between copulation and spontaneous parturition) was not altered by PQ either. Although two stillbirths from one litter were recorded, the median litter size on postnatal day 1 (PND1) did not differ between control and PQ-treated groups (Table 2). The increased proportion (20%) of treated pregnant rats (with implantation sites) not delivering viable offspring indicated that PQ caused a higher incidence of pregnancy losses (Table 2). The mean birthweight of pups exposed in utero to PQ did not differ from that of those of the unexposed control group (Table 2).

Overall, these findings indicated that PQ, at an oral dose as high as 20 mg.kg·bw^−1^·d^−1^ given throughout rat pregnancy, caused no discernible maternal toxicity and, except for a small increase in pregnancy losses, induced no developmental toxic effect (detectable at birth) either.

**Figure 4:**
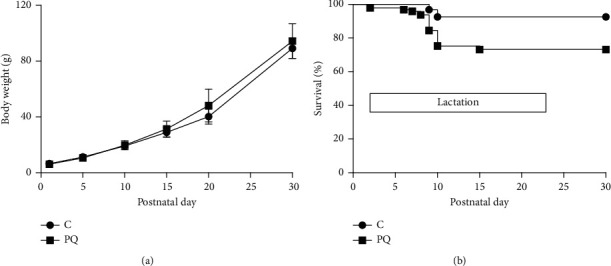
(a) Postnatal body weight gain (g) of controls (*C*) and prenatally exposed (PQ) pups (mean ± SD). (b) Kaplan–Meyer plot for % survival of control (94 pups, 11 litters) and prenatally exposed (97 pups, 14 litters) offspring up to PND 30. 26 pups (from 5 litters) born to PQ-treated pregnant females died on PNDs 2, 6, 7, 8, 9, 19, and 15, whereas 7 pups (from 4 litters) born to control females died on PNDs 9 and 10. In log-rank (Mantel–Cox) test, survival curves differ at *p*=0.0003.

During postnatal growth, 26 pups born to PQ-treated dams died between PNDs 2 and 15, compared to 7 deaths recorded among pups born to control mothers, leading to higher cumulative mortality (26.8% vs 7.4%, respectively, chi-square test *p* < 0.05) (Figure 4(b)). Body weight gain of pups prenatally exposed to PQ and body weight gain of unexposed offspring were similar (Figure 4(a)) and so were the days of appearance of landmarks of somatic maturation and neurologic reflexes acquisition (Table 3).

As shown in Table 4, the open field (OF) performance of rats prenatally exposed to PQ was somewhat distinct from that of unexposed rats. The OF is one of the most widely used tests to assess exploratory activity and emotionality in rodents. The test results indicated that, compared to controls, the offspring born to PQ-treated mothers tended to exhibit (on PND 25) a decreased locomotor activity (crosses of subdivision lines), vertical exploration (rearing up), and an increased number of grooming episodes and to spend less time in the central circle. This slightly altered pattern of OF test responses is consistent with a decreased exploratory activity and an enhanced emotionality among rats exposed in utero to PQ.

In summary, except for slightly increased number of pup deaths in midlactation period, no detrimental effects of intrauterine exposure to PQ on pup postnatal development were noted. Prenatal PQ, however, resulted in a tendency to enhanced emotionality and reduced exploratory activity (OF responses) in prepubertal rat pups.

## 4. Discussion

Concerns on PQ safety have been focused on acute hemolytic anemia in susceptible individuals, rises in methemoglobinemia levels, and prolongation of QT interval and cardiac arrhythmias at overdoses. Nonetheless, the toxicological database on PQ is incomplete. PQ produced a weak to moderate positive response in genotoxicity assays including the Salmonella/microsome assay (Ames test) [20–23]. Notwithstanding the evidence of genotoxicity, no long-term carcinogenicity study was conducted nor were PQ effects on reproductive performance, fertility, and postnatal neurobehavioral development investigated. This study contributed to bridging some of these gaps in PQ toxicological database.

In humans, PQ reaches peak concentrations within 2 to 3 h of oral dosing and elimination half-life (*t*_1/2_) is nearly 7 ± 4 h [1, 10]. PQ metabolism seems to follow two distinct pathways. Most of the absorbed drug undertakes MAO-mediated oxidative deamination to carboxy-PQ, an inactive metabolite, that is further metabolized through CYPs and phase-II enzymes [24–26]. A minor proportion of PQ is oxidized by CYPs (predominantly CYP2D6, a polymorphic enzyme) to form unstable hydroxyl metabolites (e.g., 5-hydroxy-PQ) that are believed to account for drug parasiticidal activity [25–27]. Malarial patients with a CYP2D6 poor metabolizer phenotype are refractory to PQ treatment [25, 26].

Results from this study demonstrated that PQ is efficiently transferred across placenta from the mother to the embryofetal compartment so that at term pregnancy (GD 21) concentrations of the untransformed drug in the fetal plasma were half those found in the maternal plasma. The foregoing findings suggest that maternal daily treatment with PQ on GD 0–21 leads to a significant exposure of conceptuses over the whole period of intrauterine development.

Except for an increase in gestational losses (postimplantation and whole-litter losses), no other observation suggested that PQ may have impaired embryofetal development. Pup birthweight (indication of intrauterine growth), litter sizes, and neonate viability within the first week of life were unaffected by prenatal exposure to PQ. Moreover, the postnatal growth (pup weight gain) and the somatic and neurologic development of the offspring born to PQ-treated mothers did not differ from those of the offspring of untreated females. A slightly greater pup mortality in the second week of lactation (Figure 4(b)) and an enhanced emotionality response in the OF on PND 25, however, indicated that prenatal exposure to PQ affected in some way pup survival and behavior after birth. The biological significance of these effects remains obscure and so does how prenatal exposure to PQ caused them.

## 5. Conclusions

It is of note that PQ dose tested in this study (20 mg.kg·bw^−1^·d^−1^) exceeds by a wide margin those used to treat human malaria. According to FDA-approved drug package insert, dosages of PQ recommended for pediatric patients (without G6PD deficiency) are 0.25 to 0.5 mg.kg·bw^−1^·d^−1^ for 14 days [28], i.e., PQ doses 40- and 80-fold smaller than the highest nonmaternally toxic dose used in this study. In children with mild to moderate G6PD deficiency, the recommended dose of PQ is 0.75 mg.kg·bw^−1^ once a week for 8 weeks, i.e., ≈0.1 mg.kg·bw^−1^·d^−1^, a dose 200-fold smaller than the dose tested in this study.

In conclusion, findings from this study suggested that PQ use in pregnancy, at doses substantially greater than the doses recommended for malaria therapy, does not cause maternal death or toxicity but may affect offspring postnatal survival, emotional reactivity, and exploratory activity.

## Figures and Tables

**Table 1:** Signs of toxicity noted in female rats treated with a single oral or intravenous dose of primaquine (PQ) and observed for 24 h after treatment.

Dose (mg.kg·bw^−1^)	Treated rats (*N*)	Signs of toxicity other than death	Deaths+ *N* (%)
*Oral administration*			
30	2	No signs of toxicity	0
40	2	Motor hypoactivity, teeth grinding	0

*Intravenous administration*			
5	3	No	0
10	2	Neuromuscular symptoms: hyperactivity followed by coma, tremors, muscle twitches and spasms, and ataxia	1 (50%)
20	1		1 (100%)
30	2		2 (100%)

^+^Toxicity signs and deaths after intravenous administration occurred shortly after treatment, i.e., within 1-2 min at 20 or 30 mg.kg·bw^−1^ or 5 min following 10 mg.kg·bw^−1^.

**Table 2:** Effects of primaquine administered orally to rats during pregnancy (GD 0–21) on maternal and offspring variables evaluated at birth.

Treatment	Control (6 ml.kg·bw^−1^·d^−1^)	Primaquine (20 mg.kg·bw^−1^·d^−1^)	Statistical analysis
Mated females (*N*)^#^	16	21	—
Pregnant females (with implantations), *N*	11	14	—
Pregnant/mated females (%)	68.8	66.7	ns
Total of implantation sites (*N*)	110	155	—
Implantations per female (*N*)^+^	10.0 ± 2.8	11.1 ± 4.5	ns

*Maternal weight gain* (g)			
GD 0	249.3 ± 22.4	268.7 ± 28.2	ns
GD 21	323.9 ± 23.5	345.4 ± 43.0	ns
GD 21 wt–GD 0 wt (∆g)	74.6 ± 22.1	76.6 ± 23.9	ns
Pregnancy length (*d*)	22 (22–23)	22.5 (21–23)	ns
Litter size at birth (*N*)	10 (5–13)	8 (5–13)	ns
Live pups on PND 1 (*N*)	94	97	—
Stillbirths (*N*)	0	2^++^	—
Postimplantation losses per litter (*N*)^#^	1.45 ± 1.50	4.14 ± 2.68	*p* < 0.05
Whole-litter losses (*N*) (%)^+++^	0	3 (20.0)	*p* < 0.05
Sex ratio (F/M)	0.98 ± 0.18	1.17 ± 0.15	ns
Pup body weight on PND 1 (g)	6.48 ± 0.25	6.16 ± 0.44	ns

Control rats received ultrapure water (vehicle). ^#^Mating confirmed by presence of sperm in the vaginal smear. ^+^Detected after weaning when the mothers were euthanized. ^++^ from one litter. ^+++^ % = (whole-litter losses/pregnant females) ×100. Data are mean ± SD or median and range (maximum–minimum). Wherever applicable the litter was the statistical unit of analysis. Means were compared by Student's *t*-test and nonparametric data by the Mann-Whitney *U* test. ns: nonsignificant (*p* > 0.05). Postimplantation losses = implant sites (*N*) − litter size at birth (*N*).

**Table 3:** Somatic and neurodevelopmental landmarks in the offspring of female rats treated orally with primaquine during pregnancy (GD 0–21).

Treatment in pregnancy	Control (6 ml.kg·bw^−1^·d^−1^)	Primaquine (20 mg.kg·bw^−1^·d^−1^)	Statistical analysis^+^
Examined litters (*N*)	11	11	—
*Physical landmarks*			
Ear unfolding	3 (3–4)	3 (2–5)	*p* = 0.99
Fur development	8 (6–8)	7 (6–9)	*p* = 0.99
Incisor eruption	11 (10–14)	11 (9–13)	*p* = 0.62
Eye opening	16 (14–17)	15 (14–19)	*p* = 0.99
Descent of testes	17 (15–19)	18 (15–20)	*p* = 0.21
Vaginal opening	34 (27–37)	35 (29–41)	*p* = 0.24
Preputial separation	37 (31–41)	35 (33–40)	*p* = 0.24

*Reflex maturation*			
Surface righting	4 (3–7)	3.5 (3–7)	*p* = 0.02
Cliff avoidance	8 (3–13)	9 (4–13)	*p* = 0.22
Negative geotaxis	9 (5–13)	9 (4–13)	*p* = 0.22
Palmar grasp^#^	9 (3–12)	8 (6–12)	*p* = 0.28
Auditory startle	13 (12–14)	13 (12–16)	*p* = 0.31
Free-fall righting	16 (16–22)	19 (16–21)	*p* = 0.93

Data are median and range (minimum-maximum) of the day on which landmarks appeared or reflexes were acquired. ^+^Mann-Whitney *U* test. ^*#*^Loss of response. Palmar grasp was the only reflex that was present at birth and disappeared with postnatal maturation. The litter was the unit of statistical analysis. ns: nonsignificant (*p* > 0.05).

**Table 4:** Exploratory behavior (open field test) of the offspring of female rats treated orally with primaquine during pregnancy (GD 0–21) on postnatal day 25.

Maternal treatment during pregnancy	Control (6 ml.kg·bw^−1^·d^−1^)	Primaquine (20 mg.kg·bw^−1^·d^−1^)	Statistical analysis^+^
Examined litters (*N*)	11	11	—
*Male offspring*			
Rearing up (*N*)	43.8 ± 21.1	27.5 ± 12.5	*p* = 0.03
Locomotor activity (*N*)	131.8 ± 38.9	108.2 ± 34.8	*p* = 0.15
Time spent within the central circle (s)	8.7 ± 6.9	6.8 ± 4.5	*p* = 0.02
Latency to leave the central circle (s)^#^	5.9 ± 2.7	7.7 ± 3.0	*p* = 0.16
Grooming episodes (*N*)	1.6 ± 0.6	2.2 ± 0.7	*p* = 0.07
Fecal boli (*N*)	4.1 ± 1.7	4.4 ± 1.3	*p* = 0.68

*Female offspring*			
Rearing up (*N*)	34.6 ± 11.2	24.39 ± 9.6	*p* = 0.03
Locomotor activity (*N*)	115.6 ± 28.9	97.1 ± 37.4	*p* = 0.09
Time spent within the central circle (s)	9.7 ± 5.3	6.1 ± 6.1	*p* = 0.10
Latency to leave the central circle (s)^#^	7.9 ± 6.9	7.7 ± 2.8	*p* = 0.93
Grooming episodes (*N*)	1.6 ± 0.3	2.0 ± 0.7	*p* = 0.09
Fecal boli (*N*)	4.4 ± 1.8	4.3 ± 2.3	*p* = 0.68

Data are shown as means ± SD. + Student's *t*-test. ns: nonsignificant (*p* > 0.05). The litter was the unit of statistical analysis. ^*#*^Latency to leave the central circle of OF arena where rats are placed at the beginning of each test session. Time spent in the central circle does not include the initial time (latency) to leave it.

## Data Availability

The raw data used to support the findings of this study are available from the corresponding author upon request.
